# An Efficient Estimator for Moving Target Localization Using Multi-Station Dual-Frequency Radars

**DOI:** 10.3390/s17122914

**Published:** 2017-12-15

**Authors:** Jiyan Huang, Ying Zhang, Shan Luo

**Affiliations:** School of Electronic Engineering, University of Electronic Science and Technology of China, Chengdu 610054, China; zhying@uestc.edu.cn (Y.Z.); luoshan@uestc.edu.cn (S.L.)

**Keywords:** moving target localization, Cramer–Rao lower bound (CRLB), dual-frequency radar, closed-form solution, range estimation, weighted least squares estimator

## Abstract

Localization of a moving target in a dual-frequency radars system has now gained considerable attention. The noncoherent localization approach based on a least squares (LS) estimator has been addressed in the literature. Compared with the LS method, a novel localization method based on a two-step weighted least squares estimator is proposed to increase positioning accuracy for a multi-station dual-frequency radars system in this paper. The effects of signal noise ratio and the number of samples on the performance of range estimation are also analyzed in the paper. Furthermore, both the theoretical variance and Cramer–Rao lower bound (CRLB) are derived. The simulation results verified the proposed method.

## 1. Introduction

Short-range noncontact microwave radar systems have been widely used for monitoring in military, environmental, health, and commercial systems [1,2]. Both wideband [3,4,5] and dual-frequency continuous wave (CW) [6,7,8,9,10] of signals are applied for range estimation in a short-range radar. Since a stationary scatterer will reflect wideband echo, a wideband radar is prone to clutter caused by environment. A dual-frequency CW radar is the preferred solution for moving target (MT) localization because it is immune to clutter from stationary targets [6,7,8,9,10]. For a dual-frequency radar, two carrier frequencies are transmitted simultaneously, and phase difference of Doppler signals is used to estimate the range between the target and radar. Since Doppler echo is generated only by target motion, clutter from stationary targets is eliminated in a dual-frequency radar. The separation between two carrier frequencies is used to determine a maximum unambiguous range. A range estimation method based on a dual-frequency radar is designed for gesture sensing in [6]. The authors in [7] studied the performance of range estimation using a dual-frequency radar. The radar employs a receive unit consisting of a yagi antenna. Both simulation and experimental results prove the effectiveness of the designed dual-frequency radar in [7] for indoor range estimation. Subsequently, a dual-frequency radar with antenna array is constructed for three-dimensional tracking of humans [8,9]. In this radar, phase difference between various antennas is utilized to estimate the angle of arrival (AOA), while the phase difference measured at two CW frequencies is used for target ranging. Since both range and AOA measurements are obtained, the MT can be located by a single radar. Compared with a single-station radar system, a multi-station radars system may lead to higher positioning accuracy due to its better Geometric Dilution of Precision (GDOP). For a multi-station dual-frequency radars system, a noncoherent localization scheme in [10] was proposed for MT localization only using range measurements. At least three dual-frequency radars are used to locate a MT. The noncoherent localization approach is most applicable to single-target localization, where it is not necessary to require cross-range resolution [10]. A least squares (LS) estimator was used in [10] to estimate a target position. The performance of the LS method can be further improved using a weighted least squares (WLS) estimator.

In this paper, a novel localization method with closed-form solution and high positioning accuracy is proposed for a multi-station dual-frequency radars system. The proposed method extends the two-step WLS estimator from time-difference-of-arrival (TDOA) location system [11] to a dual-frequency radars system. Like [10], this paper focuses on a single MT. Range measurements estimated from multi-station dual-frequency radars are used in the proposed method for target localization. It should be noted that frequency and phase synchronization are very important for a radar system. Imperfect synchronization will result in frequency shift and phase errors. Precise frequency and phase synchronization mainly depend on the radio frequency (RF) circuit design. For signal processing, several channel calibration techniques [12,13] was used to suppress frequency shift, Inphase/Quadrature (I/Q) mismatch, and direct current (DC) offset. After channel calibration, the proposed localization method can be used to locate a MT.

Compared with the previous research studies, the main contributions of this paper are listed as follows:

(1) The effects of signal noise ratio (SNR) and the number of samples on the performance of range estimation are analyzed in the paper. The fundamental work has been done for performance analysis of dual-frequency radars in [10] where the influences of drift in frequency and I/Q mismatch were studied. SNR and the number of samples are another two important factors which will greatly affect the performance of range estimation.
(2) A novel localization method with closed-form solution and high positioning accuracy is proposed for a multi-station dual-frequency radars system. The proposed method first derives the variances of the phase measurements. Based on the derived variances, the two weighted matrices can be calculated for two-step solutions. Due to the weighted information, the proposed method can provide the better performance than the LS estimator.
(3) Performance analysis for the proposed method is presented in this paper. Both the theoretical variance and Cramer–Rao lower bound (CRLB) for the proposed method are derived in the paper. The derived CRLB can provide a benchmark for MT localization in the dual-frequency radars system to evaluate the performance of any unbiased estimator. They have not been addressed in the literature.

Section 2 briefly introduces system model. The proposed method is presented in Section 3. Section 4 derives the CRLB. In Section 5, the performance of the proposed algorithm is simulated in terms of the root mean square error (RMSE). Conclusions of this paper are given in Section 6.

## 2. System Model

The basic model of a dual-frequency radars system for MT localization is briefly introduced in this section. Assuming that (x,y) is the position of a MT to be estimated and the known coordinate of the ith radar in a N-Radars system is $\left(x_{i},y_{i}\right)$, denote the measurement with noise of {*} as $\left\{\overset{⏜}{*}\right\}$, the true distance between the ith Radar and MT can be modeled as [14,15]:

(1) $$r_{i}^{2}={\left(x_{i}-x\right)}^{2}+{\left(y_{i}-y\right)}^{2}=k_{i}-2x_{i}x-2y_{i}y+k$$

where $k_{i}=x_{i}^{2}+y_{i}^{2}$ and $k=x^{2}+y^{2}$. The range estimate ${\overset{⏜}{r}}_{i}$ of $r_{i}$ can be obtained using a dual-frequency radar. For the ith radar, it operates at two CW frequencies $f_{i1}$ and $f_{i2}$, where both CW frequencies are combined and transmitted simultaneously. The frequency separation $f_{i2}-f_{i1}$ is used to determine a maximum unambiguous range. Assuming that $\phi_{i1}$ and $\phi_{i2}$ are the phases corresponding to two CW frequencies of operation, the range estimate can be obtained using the phase difference $\phi_{i2}-\phi_{i1}$ of the two CW frequencies [7,8]:

(2) $$r_{i}=\frac{\left(\phi_{i2}-\phi_{i1}\right)c}{4\pi \left(f_{i2}-f_{i1}\right)}=a_{i}\left(\phi_{i2}-\phi_{i1}\right)$$

where $a_{i}=c/\left(4\pi \left(f_{i2}-f_{i1}\right)\right)$. Note that the phase difference is modulo 2π, the maximum unambiguous range is [7,8]:

(3) $$r_{\max i}=\frac{c}{2\left(f_{i2}-f_{i1}\right)}$$

which depends on the frequency separation $f_{i2}-f_{i1}$. Proper selection of $f_{i2}-f_{i1}$ can provide the sufficient scope of unambiguous range. Compared with $r_{\max i}=3.75\;\mathrm{cm}$ for a radar with single carrier frequency, the maximum unambiguous range increases to 100 m when the frequency separation is set to be 1.5 MHz in a dual-frequency radar.

## 3. Closed-Form Solution for Dual-Frequency Radars

Although a LS estimator was utilized in [10] for MT location in a dual-frequency radars system, the performance of the LS estimator can be further improved using a WLS estimator. This section proposes a novel localization method with a closed-form solution based on a two-step WLS estimator for dual-frequency radars. The proposed method starts with baseband signals which may help to further understand the dual-frequency radar. The theoretical variance of the proposed method is also derived in this section.

### 3.1. Performance Analysis of Range Estimation Using FFT

Obviously, the positioning accuracy of MT depends on the estimate error of ${\overset{⏜}{r}}_{i}$. This subsection analyzes the effects of SNR and the number of samples on the performance of range estimation. It is observed from Equation (2) that the range estimate ${\overset{⏜}{r}}_{i}$ relies on the phase parameters ${\overset{⏜}{\phi }}_{i1}$ and ${\overset{⏜}{\phi }}_{i2}$. Hence, ${\overset{⏜}{\phi }}_{i1}$ and ${\overset{⏜}{\phi }}_{i2}$ should be obtained firstly. In this subsection, the phase parameters are estimated using the fast Fourier transformation (FFT) method since it is widely used in phase estimation [8,9] and it can attain the satisfactory performance even in low SNR situation (SNR≤0 dB) with sufficient samples. Furthermore, the theoretical variances of ${\overset{⏜}{\phi }}_{i1}$ and ${\overset{⏜}{\phi }}_{i2}$ are derived to evaluate the performance of the phase estimation. They are also used for the weighted matrix in the proposed location method, which is shown in Section 3.2.

For the ith dual-frequency radar, the baseband signals corresponding to each carrier frequency can be expressed as:

(4) $${\overset{⏜}{U}}_{il}\left(k\right)=U_{il}\left(k\right)+n_{il}\left(k\right),k=1,\cdots ,M,l=1,2$$

where k is the time index, M is the number of samples, and $U_{il}\left(k\right)=UI_{il}\left(k\right)+jUQ_{il}\left(k\right)$ is the complex signal of the baseband echo. Without loss of generality, the amplitude of $U_{il}\left(k\right)$ can be normalized to 1. Hence, $U_{il}\left(k\right)$ can be rewritten as:

(5) $${\overset{⏜}{U}}_{il}\left(k\right)=e^{j\phi_{il}^{\prime }\left(k\right)}+n_{il}\left(k\right)=e^{j\left(2\pi fd_{il}\left(k-1\right)/fs+\phi_{il}\right)}+n_{il}\left(k\right),k=1,\cdots ,M,l=1,2$$

where $\phi_{il}^{\prime }\left(k\right)=2\pi fd_{il}\left(k-1\right)/f_{s}+\phi_{il}$, $fd_{il}$ is Doppler frequency, fs is sampling frequency, and $n_{il}\left(k\right)=nI_{il}\left(k\right)+jnQ_{il}\left(k\right)$ is a Gaussian noise with zero mean and variance $\sigma_{ni}^{2}$. Since I/Q channel has the independent analog digital converter (ADC), the following equations holds:

(6) $$E\left[nI_{il}\left(k\right)\right]=E\left[nQ_{il}\left(k\right)\right]=E\left[nI_{il}\left(k\right)nQ_{il}\left(k\right)\right]=0.$$

Obviously, the SNR of baseband signals received by the ith radar is:

(7) $$SNR_{i}=\frac{1}{\sigma_{ni}^{2}}.$$

The Fourier transform of (4) is:

(8) $$Uf_{il}\left(k_{f}\right)=\sum_{k=1}^{M}U_{il}\left(k\right)W\left(k\right)$$

where $k_{f}$ is the frequency index corresponding to Doppler shift, $W\left(k\right)=e^{-2\pi (k-1)(k_{f}-1)/M}$ is an Mth root of unity.

Substituting $U_{il}\left(k\right)=UI_{il}\left(k\right)+jUQ_{il}\left(k\right)$ and W(k)=WI(k)+jWQ(k) into (8) gives:

(9) $$Uf_{il}\left(k_{f}\right)=\sum_{k=1}^{M}\left(UI_{il}\left(k\right)+jUQ_{il}\left(k\right)\right)\left(WI\left(k\right)+jWQ\left(k\right)\right)=\sum_{k=1}^{M}VI_{il}\left(k\right)+j\sum_{k=1}^{M}VQ_{il}\left(k\right)$$

where

(10) $$VI_{il}\left(k\right)=UI_{il}\left(k\right)WI\left(k\right)-UQ_{il}\left(k\right)WQ\left(k\right)$$

(11) $$VQ_{il}\left(k\right)=UI_{il}\left(k\right)WQ\left(k\right)+UQ_{il}\left(k\right)WI\left(k\right).$$

From (9), the phase $\phi_{il}$ can be obtained:

(12) $$\phi_{il}=a\tan\frac{\sum_{k=1}^{M}VQ_{il}\left(k\right)}{\sum_{k=1}^{M}VI_{il}\left(k\right)}$$

The estimate variance of $\phi_{il}$ can be calculated by using the perturbation approach. Further, Δ is denoted as error perturbation. In presence of noise and disturbance, $\Delta \phi_{il}$ can be obtained using differential scheme:

(13) $$\Delta \phi_{il}=\frac{\sum_{k=1}^{M}\Delta VQ_{il}\left(k\right)\sum_{k=1}^{M}VI_{il}\left(k\right)-\sum_{k=1}^{M}VQ_{il}\left(k\right)\sum_{k=1}^{M}\Delta VI_{il}\left(k\right)}{{\left(\sum_{k=1}^{M}VI_{il}\left(k\right)\right)}^{2}+{\left(\sum_{k=1}^{M}VQ_{il}\left(k\right)\right)}^{2}}=\frac{\sum_{k=1}^{M}\Delta VQ_{il}\left(k\right)\sum_{k=1}^{M}VI_{il}\left(k\right)-\sum_{k=1}^{M}VQ_{il}\left(k\right)\sum_{k=1}^{M}\Delta VI_{il}\left(k\right)}{{\left|Uf_{il}\left(k_{f}\right)\right|}^{2}}$$

where

(14) $$\Delta VI_{il}\left(k\right)=\Delta UI_{il}\left(k\right)WI\left(k\right)-\Delta UQ_{il}\left(k\right)WQ\left(k\right)=nI_{il}\left(k\right)WI\left(k\right)-nQ_{il}\left(k\right)WQ\left(k\right)$$

(15) $$\Delta VQ_{il}\left(k\right)=\Delta UI_{il}\left(k\right)WQ\left(k\right)+\Delta UQ_{il}\left(k\right)WI\left(k\right)=nI_{il}\left(k\right)WQ\left(k\right)+nQ_{il}\left(k\right)WI\left(k\right).$$

It should be noted that ${\left|Uf_{il}\left(k_{f}\right)\right|}^{2}$ is power spectrum of $U_{il}\left(k\right)$ at the Doppler frequency $k_{f}$. For a single MT, ${\left|Uf_{il}\left(k_{f}\right)\right|}^{2}=M^{2}$.

Substituting ${\left|Uf_{il}\left(k_{f}\right)\right|}^{2}=M^{2}$ into (13) gives:

(16) $$\Delta \phi_{il}=\frac{\sum_{k=1}^{M}\Delta VQ_{il}\left(k\right)\sum_{k=1}^{M}VI_{il}\left(k\right)-\sum_{k=1}^{M}VQ_{il}\left(k\right)\sum_{k=1}^{M}\Delta VI_{il}\left(k\right)}{M^{2}}$$

Noting that $WI{\left(k\right)}^{2}+WQ{\left(k\right)}^{2}=1$ and deriving from (6), (14) and (15), we have:

(17) $$E\left[\Delta VI_{il}\left(k\right)\right]=E\left[\Delta VQ_{il}\left(k\right)\right]=E\left[\Delta VI_{il}\left(k\right)\Delta VQ_{il}\left(k\right)\right]=0$$

(18) $$E\left[\Delta VI_{il}{\left(k\right)}^{2}\right]=E\left[\Delta VQ_{il}{\left(k\right)}^{2}\right]=\frac{\sigma_{ni}^{2}}{2}.$$

It can be derived from (16)–(18) that:

(19) $$E\left[\Delta \phi_{il}\right]=0$$

(20) $$\mathrm{cov}\left(\Delta \phi_{il}\right)=E\left[\Delta \phi_{il}{}^{2}\right]=\frac{\sigma_{ni}^{2}}{2M}=\frac{1}{2MSNR_{i}}.$$

From (2), $\Delta r_{i}$ is derived as:

(21) $$\Delta r_{i}=\frac{\left(\Delta \phi_{i2}-\Delta \phi_{i1}\right)c}{4\pi \left(f_{i2}-f_{i1}\right)}.$$

It can be derived from (19)–(21) that the mean and variance of $\Delta r_{i}$ are:

(22) $$E\left[\Delta r_{i}\right]=\left(E\left[\Delta \phi_{i2}\right]-E\left[\Delta \phi_{i1}\right]\right)a_{i}=0$$

(23) $$\mathrm{cov}\left(\left[\Delta r_{i}\right]\right)=E\left[{\left(\Delta r_{i}\right)}^{2}\right]=a_{i}^{2}\left(E\left[{\left(\Delta \phi_{i2}\right)}^{2}\right]-2E\left[\Delta \phi_{i2}\right]E\left[\Delta \phi_{i1}\right]+E\left[{\left(\Delta \phi_{i2}\right)}^{2}\right]\right)=\frac{a_{i}^{2}}{MSNR_{i}}=\frac{c^{2}}{MSNR_{i}{\left(4\pi \left(f_{i2}-f_{i1}\right)\right)}^{2}}.$$

It can be seen from (22) and (23) that the FFT method is an unbiased estimator and it depends on SNR and the number of samples. The accuracy of range estimation can be improved through increasing SNR and the number of samples. Equation (23) also implies that the smaller frequency separation will lead to larger range estimation error. Thus, a proper selection of frequency separation should consider both the maximum unambiguous range and the accuracy of range estimation.

### 3.2. Closed-Form Solution for Multi-Station Dual-Frequency Radars System

In this subsection, a novel localization scheme based on the two-step WLS estimator for a multi-station dual-frequency radars system is proposed.

Substituting (2) into (1) gives:

(24) $$2x_{i}x+2y_{i}y-k=k_{i}-\left(\phi_{i2}^{2}-2\phi_{i2}\phi_{i1}+\phi_{i1}^{2}\right)a_{i}^{2}.$$

With the measurement noise, the error vector derived from (24) is:

(25) e=Y−GZ

where

$$Y=\left[\begin{array}{c} k_{1}-\left({\overset{⏜}{\phi }}_{12}^{2}-2{\overset{⏜}{\phi }}_{12}{\overset{⏜}{\phi }}_{11}+{\overset{⏜}{\phi }}_{11}^{2}\right)a_{1}^{2}\\ \vdots \\ k_{N}-\left({\overset{⏜}{\phi }}_{N2}^{2}-2{\overset{⏜}{\phi }}_{N2}{\overset{⏜}{\phi }}_{N1}+{\overset{⏜}{\phi }}_{N1}^{2}\right)a_{N}^{2} \end{array}\right],\;G=\left[\begin{array}{ccc} 2x_{1} & 2y_{1} & -1\\ \vdots & \vdots & \vdots \\ 2x_{N} & 2y_{N} & -1 \end{array}\right],\;Z={\left[\begin{array}{ccc} x & y & k \end{array}\right]}^{T}.$$

The first step WLS estimator of Z can be obtained from (25):

(26) $$Z=\arg\min\left\{{\left(Y-GZ\right)}^{T}\Psi^{-1}\left(Y-GZ\right)\right\}={\left(G^{T}\Psi^{-1}G\right)}^{-1}G^{T}\Psi^{-1}Y$$

where ψ is the covariance matrix of e:

(27) $$\psi =\mathrm{cov}\left(e\right)=E\left(ee^{T}\right).$$

Ignoring the square error term and derived from (24), the element $e_{i}$ of e can be expressed as:

(28) $$e_{i}=-2a_{i}^{2}\left(\phi_{i2}-\phi_{i1}\right)\left(\Delta \phi_{i2}-\Delta \phi_{i1}\right).$$

The expectation of $e_{i}e_{j}$ can be derived from (19), (20) and (28):

(29) $$E\left(e_{i}e_{j}\right)=\{\begin{array}{c} \begin{array}{cc} 0 & i\neq j \end{array}\\ \begin{array}{cc} 4a_{i}^{4}{\left(\phi_{i2}-\phi_{i1}\right)}^{2}\frac{1}{MSNR_{i}} & i=j \end{array} \end{array}.$$

Based on (29), Equation (27) can be decomposed as:

(30) $$\psi =\mathrm{cov}\left(e\right)=E\left(ee^{T}\right)=BQB$$

where

(31) $$B=diag\left\{\left[\begin{array}{ccc} 2a_{1}^{2}\left(\phi_{12}-\phi_{11}\right) & \cdots & 2a_{N}^{2}\left(\phi_{N2}-\phi_{N1}\right) \end{array}\right]\right\}$$

(32) $$Q=diag\left\{\left[\begin{array}{ccc} \frac{1}{MSNR_{1}} & \cdots & \frac{1}{MSNR_{N}} \end{array}\right]\right\}.$$

Since the covariance matrix ψ depends on the unknown $\phi_{i1}$ and $\phi_{i2}$, the approximate values ${\overset{⏜}{\phi }}_{i1}$ and ${\overset{⏜}{\phi }}_{i2}$ can be used in ψ to make the problem solvable.

The first step solution of Z in (26) is based on the assumption of independent x, y, and k. However, those parameters are correlated by $k=x^{2}+y^{2}$. The estimation accuracy can be further improved using the relationship between x, y, and k. The results can be revised as follows using the relation of $k=x^{2}+y^{2}$:

(33) $$e^{\prime }=Y^{\prime }-G^{\prime }Z^{\prime }$$

where $e^{\prime }={\left[\begin{array}{ccc} e_{1}^{\prime } & e_{2}^{\prime } & e_{3}^{\prime } \end{array}\right]}^{T}$ is the error vector,

$$Y^{\prime }=\left[\begin{array}{c} Z_{1}^{2}\\ Z_{2}^{2}\\ Z_{3} \end{array}\right],\;G^{\prime }=\left[\begin{array}{cc} 1 & 0\\ 0 & 1\\ 1 & 1 \end{array}\right],\;Z^{\prime }=\left[\begin{array}{c} x^{2}\\ y^{2} \end{array}\right].$$

It can be seen from (33) that $Z^{\prime }={\left[\begin{array}{cc} x^{2} & y^{2} \end{array}\right]}^{T}$. To obtain the MT position (x,y), $Z^{\prime }$ in (33) should be solved first.

$Z^{\prime }$ can be obtained from (33) using the second step WLS solution:

(34) $$Z^{\prime }={\left(G^{\prime }{}^{T}\Psi^{\prime }{}^{-1}G^{\prime }\right)}^{-1}G^{\prime }{}^{T}\Psi^{\prime }{}^{-1}Y^{\prime }$$

where $\Psi^{\prime }$ is the covariance matrix of $e^{\prime }$. The final estimation of the MS position $Z^{\prime \prime }={\left[\begin{array}{cc} x & y \end{array}\right]}^{T}$ is:

(35) $$Z^{\prime \prime }=sign\left(Z\right)\sqrt{Z^{\prime }}.$$

Using the perturbation approach as (28) and [11], the covariance matrix of $Z^{\prime \prime }$ can be obtained from (35):

(36) $$\mathrm{cov}\left(Z^{\prime \prime }\right)=B^{\prime \prime }{}^{-1}\mathrm{cov}{\left(Z^{\prime }\right)}_{2\times 2}B^{\prime \prime }{}^{-1}$$

where $\mathrm{cov}\left(Z^{\prime }\right)={\left(G^{\prime }{}^{T}\Psi^{\prime }{}^{-1}G^{\prime }\right)}^{-1}$, $\Psi^{\prime }=E\left(e^{\prime }e^{\prime }{}^{T}\right)=B^{\prime }\mathrm{cov}\left(Z\right)B^{\prime }$, $\mathrm{cov}\left(Z\right)={\left(G^{T}\Psi^{-1}G\right)}^{-1}$, $B^{\prime \prime }=diag\left\{\left[\begin{array}{cc} 2x & 2y \end{array}\right]\right\}$, and $B^{\prime }=diag\left\{\left[2x,2y,1\right]\right\}$.

## 4. Cramer–Rao Lower Bound

It is well known that the CRLB sets a lower limit for the variance or covariance matrix of any unbiased estimate of unknown parameters [16]. This subsection derives the CRLB for MT localization in a dual-frequency radars system, which can provide a benchmark to evaluate the performance of any unbiased estimator.

Let $\overset{⏜}{U}$ be a measurement vector which contains ${\overset{⏜}{U}}_{i1}\left(k\right)$, ${\overset{⏜}{U}}_{i2}\left(k\right)$, i=1,⋯,N and k=1,⋯,M. Note that $\phi_{i2}=r_{i}/a_{i}+\phi_{i1}$, the unknown parameter vector θ is:

(37) $${\left[\begin{array}{ccccccccccc} x & y & \phi_{11} & \cdots & \phi_{N1} & fd_{11} & \cdots & fd_{N1} & fd_{12} & \cdots & fd_{N2} \end{array}\right]}^{T}.$$

The CRLB matrix is defined as the inverse of the Fisher information matrix (FIM) $J_{\theta }$:

(38) $$E\left(\left(\overset{⏜}{\theta }-\theta \right){\left(\overset{⏜}{\theta }-\theta \right)}^{T}\right)\geq J_{\theta }^{-1}$$

where $\overset{⏜}{\theta }$ is an estimate of θ.

The FIM is determined by [16]:

(39) $$J_{\theta }=E\left[\frac{\partial \ln f\left(\overset{⏜}{U}|\theta \right)}{\partial \theta }{\left(\frac{\partial \ln f\left(\overset{⏜}{U}|\theta \right)}{\partial \theta }\right)}^{T}\right].$$

From (5), the probability density function (PDF) $f\left(\overset{⏜}{U}|\theta \right)$ can be written as:

(40) $$f\left(\overset{⏜}{U}|\theta \right)=\prod_{i=1}^{N}\prod_{k=1}^{M}f\left({\overset{⏜}{U}}_{i1}\left(k\right)\right)f\left({\overset{⏜}{U}}_{i2}\left(k\right)\right)$$

where

(41) $$f\left({\overset{⏜}{U}}_{il}\left(k\right)\right)=f\left(\overset{⏜}{U}I_{il}\left(k\right)\right)f\left(\overset{⏜}{U}Q_{il}\left(k\right)\right),l=1,2$$

with

(42) $$f\left(\overset{⏜}{U}I_{il}\left(k\right)\right)\propto \exp\left(-\frac{{\left(\overset{⏜}{U}I_{il}\left(k\right)-\cos\phi_{il}^{\prime }\left(k\right)\right)}^{2}}{\sigma_{ni}^{2}}\right)=\exp\left(-\frac{{\left(\overset{⏜}{U}I_{il}\left(k\right)-\cos\left(2\pi fd_{il}\left(k-1\right)/fs+\phi_{il}\right)\right)}^{2}}{\sigma_{ni}^{2}}\right)$$

(43) $$f\left(\overset{⏜}{U}Q_{il}\left(k\right)\right)\propto \exp\left(-\frac{{\left(\overset{⏜}{U}Q_{il}\left(k\right)-\sin\phi_{il}^{\prime }\left(k\right)\right)}^{2}}{\sigma_{ni}^{2}}\right)=\exp\left(-\frac{{\left(\overset{⏜}{U}Q_{il}\left(k\right)-\sin\left(2\pi fd_{il}\left(k-1\right)/fs+\phi_{il}\right)\right)}^{2}}{\sigma_{ni}^{2}}\right)$$

Substituting (40)–(43) into $\partial \ln f\left(\overset{⏜}{U}|\theta \right)/\partial \theta$ gives:

(44) $$\frac{\partial \ln f\left(\overset{⏜}{U}|\theta \right)}{\partial \theta }=Hn$$

where

(45) $$H={\left[\begin{array}{ccccc} H_{x}^{T} & H_{y}^{T} & H_{\phi 1}^{T} & H_{fd1}^{T} & H_{fd2}^{T} \end{array}\right]}^{T}$$

with

$\frac{\partial r_{i}}{\partial x}=\frac{x-x_{i}}{r_{i}}$, $\frac{\partial r_{i}}{\partial y}=\frac{y-y_{i}}{r_{i}}$
$H_{x}=\left[\begin{array}{ccc} H_{x1} & \cdots & H_{xN} \end{array}\right]$
$H_{xi}=\left[\begin{array}{cccccccc} 0 & 0 & \frac{2}{a_{i}\sigma_{ni}^{2}}\frac{\partial r_{i}}{\partial x} & \frac{2}{a_{i}\sigma_{ni}^{2}}\frac{\partial r_{i}}{\partial x} & 0 & 0 & 0 & 0 \end{array}\right]$
$H_{y}=\left[\begin{array}{ccc} H_{y1} & \cdots & H_{yN} \end{array}\right]$
$H_{yi}=\left[\begin{array}{cccccccc} 0 & 0 & \frac{2}{a_{i}\sigma_{ni}^{2}}\frac{\partial r_{i}}{\partial y} & \frac{2}{a_{i}\sigma_{ni}^{2}}\frac{\partial r_{i}}{\partial y} & 0 & 0 & 0 & 0 \end{array}\right]$
$H_{\phi 1}={\left[\begin{array}{ccc} H_{\phi 1\_1}^{T} & \cdots & H_{\phi 1\_N}^{T} \end{array}\right]}^{T}$
$H_{\phi 1\_i}=\left[\begin{array}{cccccccc} 0 & \cdots & 1 & 1 & 1 & 1 & \cdots & 0 \end{array}\right]\times \frac{2}{\sigma_{ni}^{2}}$, where the starting index of 1 in $H_{\phi 1\_i}$ is (i−1)8+1.
$H_{fdl}={\left[\begin{array}{ccc} H_{fdl\_1}^{T} & \cdots & H_{fdl\_N}^{T} \end{array}\right]}^{T},l=1,2$
$H_{fdl\_i}=\left[\begin{array}{cccccc} 0 & \cdots & 1 & 1 & \cdots & 0 \end{array}\right]\times \frac{4\pi }{\sigma_{ni}^{2}fs}$, where the starting index of 1 in $H_{fdl\_i}$ is (i−1)8+4+2(l−1)+1.
$n={\left[\begin{array}{ccc} n_{1}^{T} & \cdots & n_{N}^{T} \end{array}\right]}^{T}$ is the noise vector and $n_{i}={\left[\begin{array}{cc} n_{i1}^{T} & n_{i2}^{T} \end{array}\right]}^{T}$

where

(46) $$n_{i1}={\left[\begin{array}{cccc} \sum_{k=1}^{M}\left\{-\sin\phi_{i1}^{\prime }\left(k\right)nI_{i1}\left(k\right)\right\} & \sum_{k=1}^{M}\left\{\cos\phi_{i1}^{\prime }\left(k\right)nQ_{i1}\left(k\right)\right\} & \sum_{k=1}^{M}\left\{-\sin\phi_{i2}^{\prime }\left(k\right)nI_{i2}\left(k\right)\right\} & \sum_{k=1}^{M}\left\{\cos\phi_{i2}^{\prime }\left(k\right)nQ_{i2}\left(k\right)\right\} \end{array}\right]}^{T}$$

(47) $$\begin{array}{c} n_{i2}=[\begin{array}{cc} \sum_{k=1}^{M}\left\{-\sin\phi_{i1}^{\prime }\left(k\right)\left(k-1\right)nI_{i1}\left(k\right)\right\} & \sum_{k=1}^{M}\left\{\cos\phi_{i1}^{\prime }\left(k\right)\left(k-1\right)nQ_{i1}\left(k\right)\right\} \end{array}\\ {\begin{array}{cc} \sum_{k=1}^{M}\left\{-\sin\phi_{i2}^{\prime }\left(k\right)\left(k-1\right)nI_{i2}\left(k\right)\right\} & \sum_{k=1}^{M}\left\{\cos\phi_{i2}^{\prime }\left(k\right)\left(k-1\right)nQ_{i2}\left(k\right)\right\} \end{array}]}^{T} \end{array}$$

The covariance matrix of $n_{i}$ is derived as

(48) $$E\left[n_{i}n_{i}^{T}\right]=A_{i}=\left[\begin{array}{cc} A_{i11} & A_{i12}\\ A_{i21} & A_{i22} \end{array}\right]=\left[\begin{array}{cc} E\left[n_{i1}n_{i1}^{T}\right] & E\left[n_{i1}n_{i2}^{T}\right]\\ E\left[n_{i2}n_{i1}^{T}\right] & E\left[n_{i2}n_{i2}^{T}\right] \end{array}\right]$$

with

$$E\left[n_{i1}n_{i1}^{T}\right]=\frac{\sigma_{ni}^{2}}{2}diag\left\{\left[\begin{array}{cccc} \sum_{k=1}^{M}{\left(\sin\phi_{i1}^{\prime }\left(k\right)\right)}^{2} & \sum_{k=1}^{M}{\left(\cos\phi_{i1}^{\prime }\left(k\right)\right)}^{2} & \sum_{k=1}^{M}{\left(\sin\phi_{i2}^{\prime }\left(k\right)\right)}^{2} & \sum_{k=1}^{M}{\left(\cos\phi_{i2}^{\prime }\left(k\right)\right)}^{2} \end{array}\right]\right\}$$

$$\begin{array}{c} E\left[n_{i2}n_{i1}^{T}\right]=E\left[n_{i1}n_{i2}^{T}\right]=\frac{\sigma_{ni}^{2}}{2}diag\{[\begin{array}{cc} \sum_{k=1}^{M}{\left(\sin\phi_{i1}^{\prime }\left(k\right)\right)}^{2}\left(k-1\right) & \sum_{k=1}^{M}{\left(\cos\phi_{i1}^{\prime }\left(k\right)\right)}^{2}\left(k-1\right) \end{array}\\ \begin{array}{cc} \sum_{k=1}^{M}{\left(\sin\phi_{i2}^{\prime }\left(k\right)\right)}^{2}\left(k-1\right) & \sum_{k=1}^{M}{\left(\cos\phi_{i2}^{\prime }\left(k\right)\right)}^{2}\left(k-1\right) \end{array}]\} \end{array}$$

$$\begin{array}{c} E\left[n_{i2}n_{i2}^{T}\right]=\frac{\sigma_{ni}^{2}}{2}diag\{[\begin{array}{cc} \sum_{k=1}^{M}{\left(\left(k-1\right)\sin\phi_{i1}^{\prime }\left(k\right)\right)}^{2} & \sum_{k=1}^{M}{\left(\left(k-1\right)\cos\phi_{i1}^{\prime }\left(k\right)\right)}^{2} \end{array}\\ \begin{array}{cc} \sum_{k=1}^{M}{\left(\left(k-1\right)\sin\phi_{i2}^{\prime }\left(k\right)\right)}^{2} & \sum_{k=1}^{M}{\left(\left(k-1\right)\cos\phi_{i2}^{\prime }\left(k\right)\right)}^{2} \end{array}]\} \end{array}$$

The expectation of $n_{i}n_{j}^{T}$ is:

(49) $$E\left[n_{i}n_{j}^{T}\right]=E\left[n_{i}\right]E\left[n_{j}^{T}\right]=0,i\neq j.$$

The covariance matrix of n can be obtained from (48) and (49):

(50) $$W=E\left[nn^{T}\right]=diag\left\{\left[\begin{array}{ccc} A_{1} & \cdots & A_{N} \end{array}\right]\right\}.$$

Substituting (44) and (50) into (39) gives:

(51) $$J_{\theta }=HWH^{T}.$$

Finally, the proposed CRLB is derived as:

(52) $$CRLB_{x,y}={\left[{\left(HWH^{T}\right)}^{-1}\right]}_{2\times 2}.$$

## 5. Simulation Results

In the simulations, the carrier frequency of radars is 24 GHz and frequency separation is 1.5 MHz. This means the maximum unambiguous range is 100 m. The Zero-IF technique is used to reduce the sampling frequency and eliminate clutter from stationary targets. The sampling frequency is set to be 10 Khz, which is sufficient to cover the Doppler shift caused by the target motion.

### 5.1. Range Estimation

**Figure 1:**
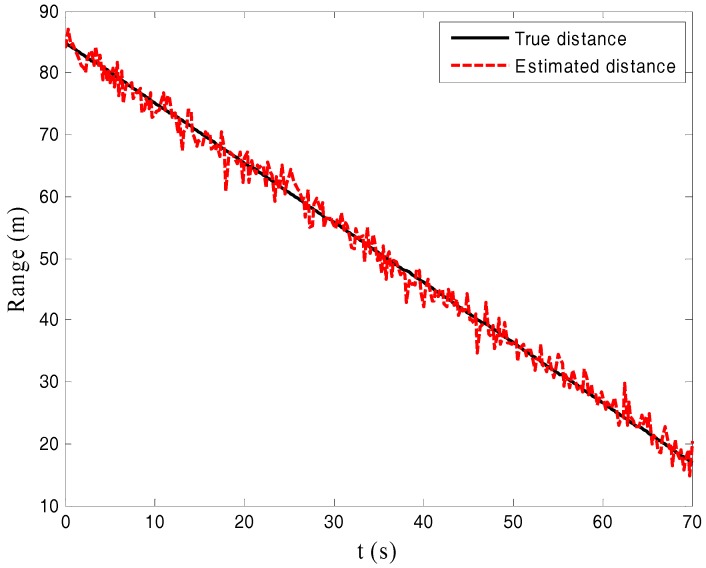
True trace and the estimated range.

This subsection is simulated to study the effects of SNR and the number of samples on the performance of range estimation. Uniform motion in [10] is used in this simulation. Consider a single-point target moving towards the origin with uniform velocity $v_{0}$ where $r=r_{0}-v_{0}t$. The speed of target is set to be 3.5 km/h. Figure 1 shows the true trace and the estimated range when the number of samples is M = 256 and SNR = −5 dB. It can be seen from this figure that the FFT method can provide the accurate range estimate even in low SNR situation (SNR < 0 dB).

**Figure 2:**
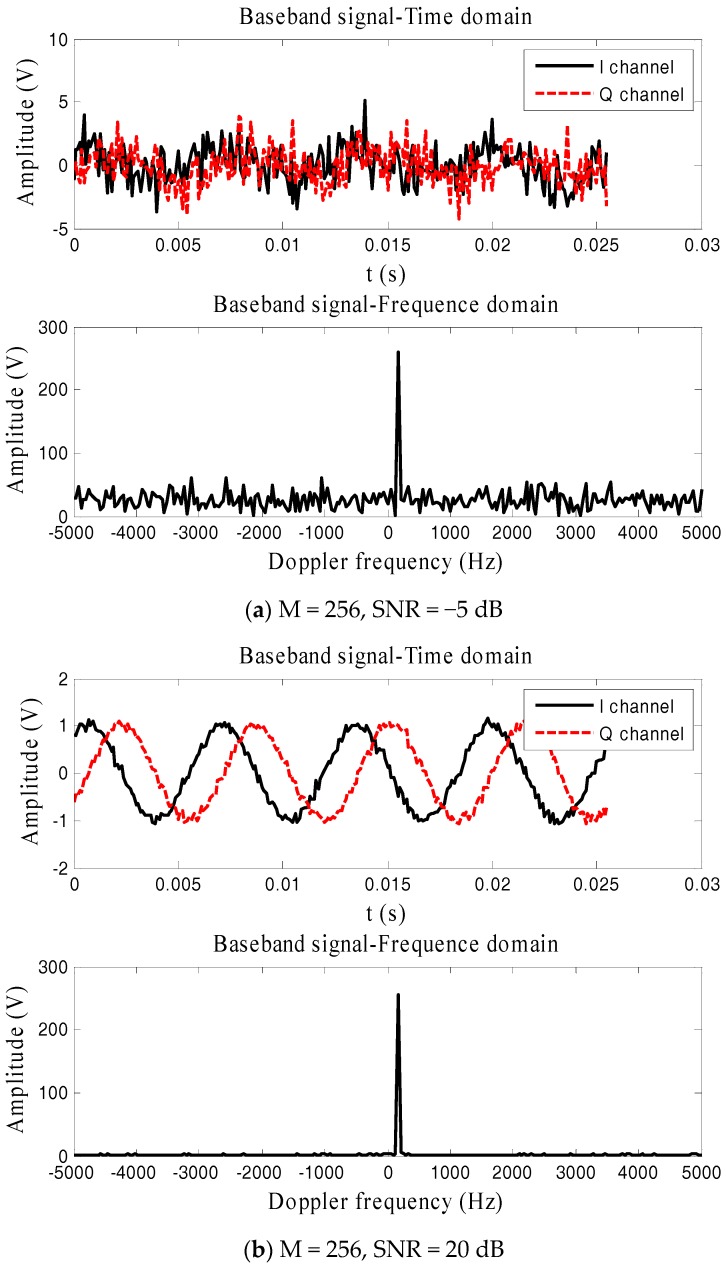
Baseband data in time domain and frequency domain.

Baseband signals in time domain and frequency domain for SNR = −5 dB and SNR = 20 dB are plotted in Figure 2. For the case with SNR = −5 dB, the time domain signals sink below the measurement noise whereas the FFT can obtain the M times gain in the frequency domain. This implies that FFT is an effective method to estimate the range in a dual-frequency radars system.

**Figure 3:**
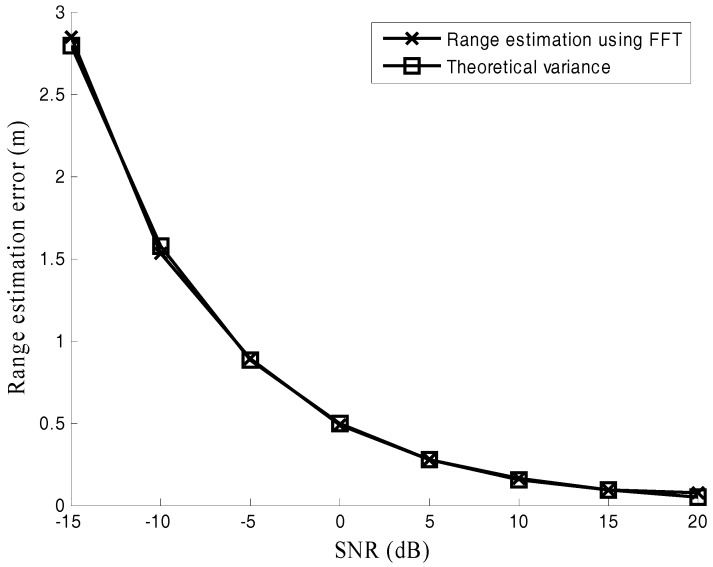
Range estimation error under different signal noise ratio (SNR).

**Figure 4:**
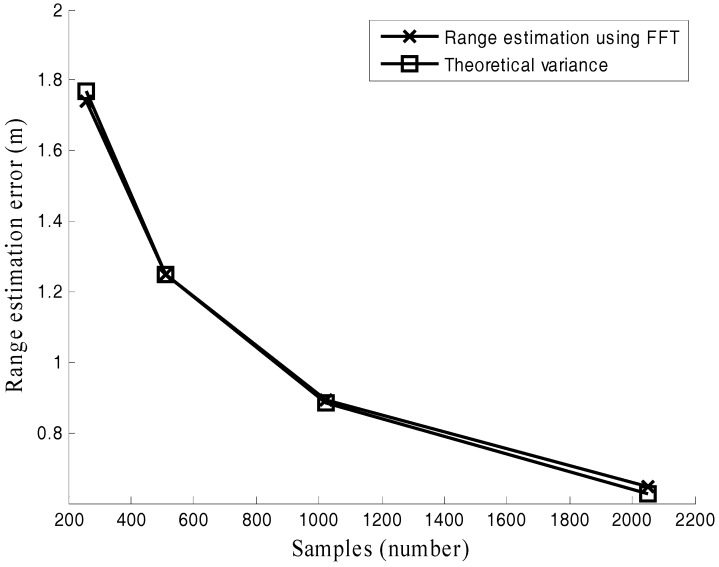
Range estimation error under different numbers of samples (M).

Both the RMSE of range estimation and its theoretical variance (23) are recorded in Figure 3 and Figure 4. Figure 3 shows the RMSEs of range estimation versus SNR when the number of samples is M = 1024. It is observed that the performance of the FFT method well matches the theoretical variance. 

Performance comparisons with different M are recorded in Figure 4. In this simulation, the number of samples M is varied from 256 to 2048 and SNR = −5 dB. Figure 4 shows that the number of samples M can help the FFT method to attain the high ranging accuracy which is in line with (23). 

Both Figure 3 and Figure 4 verify the effectiveness of the derived theoretical variance (23).

### 5.2. Target Localization

**Figure 5:**
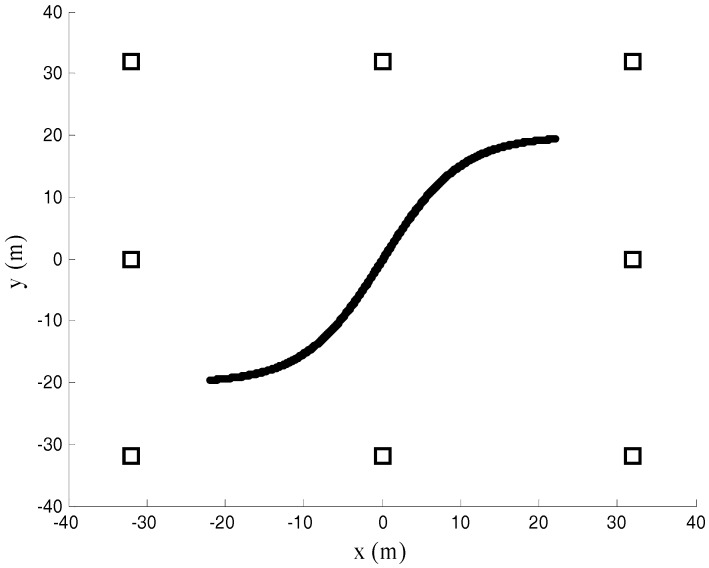
Radars layout.

A square region of dimensions 64 m × 64 m is considered for simulations. Starting with three radars with coordinates (−32, −32) m, (32, −32) m, and (32, 32) m, the radars with coordinates (−32, 32) m, (0, −32) m, (32, 0) m, (0, 32) m, and (−32, 0) m are added successively. The ‘S’ trajectory as shown in Figure 5 is used in the simulation. The time gap between the two trajectory points is 0.3 s. The radial velocity between the adjacent trajectory points is set to be 3.5 km/h. The RMSEs are defined as $\sqrt{E\left[{\left(x-\overset{⏜}{x}\right)}^{2}+{\left(y-\overset{⏜}{y}\right)}^{2}\right]}$ in the units of m.

Based on the estimated range using the FFT method, the LS localization method is used in this simulation to compare with the proposed method. The LS solution is selected here due to its closed-form solution and also because the LS estimator has been used for a dual-frequency radars system in [10]. Comparisons among the derived CRLB (52) and theoretical variance (36) of the proposed method are also given in the simulations.

**Figure 6:**
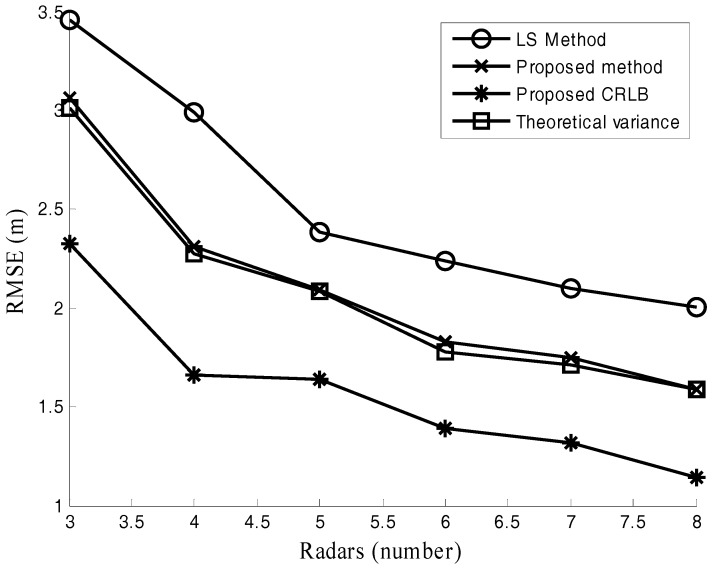
Positioning error under different numbers of radars.

**Figure 7:**
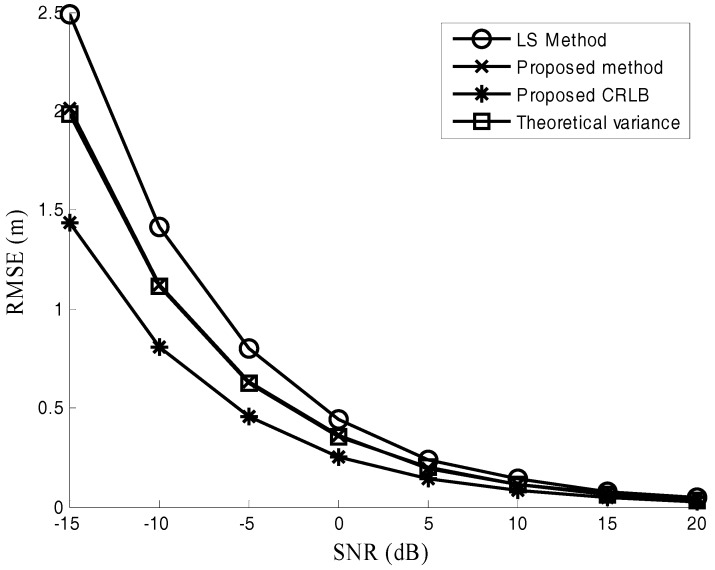
Positioning error under different SNR.

**Figure 8:**
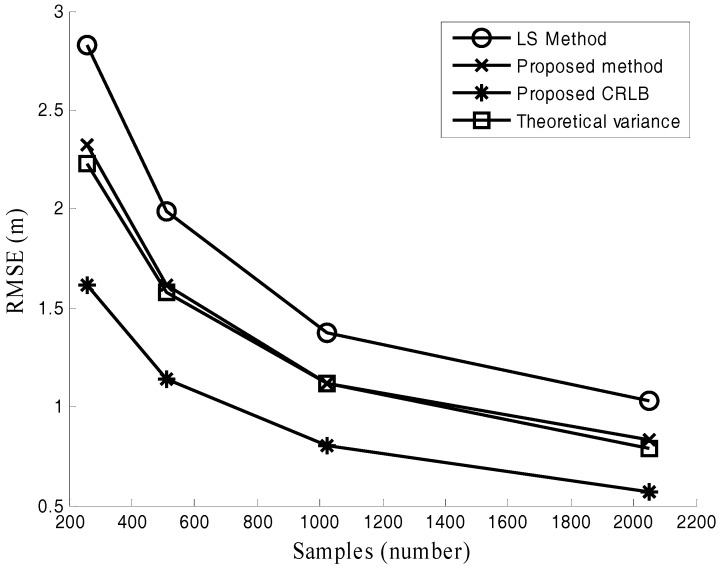
Positioning error under different M.

Figure 6, Figure 7 and Figure 8 are performed to verify the performance of the proposed location method in various channel environments. The performance under different numbers of radars is recorded in Figure 6. In this simulation, M = 512 and SNR = −10 dB. Figure 6 shows that the positioning error of the proposed method is about 0.5 m smaller than that of LS method for different numbers of radars. The positioning error versus SNR is plotted in Figure 7. In this simulation, the number of samples M = 1024. It is observed from Figure 7 that the advantage of the proposed method presents increasing trend as SNR decreases. The positioning accuracy is improved about 22% in the low SNR situations (−15 dB≤SNR≤0 dB). The effect of the different number of samples M is studied in Figure 8, where SNR = −10 dB. For the case with different numbers of samples in Figure 8, the improved positioning accuracy is about 21%.

It can be seen from Figure 6, Figure 7 and Figure 8 that the proposed method provides much better performance than the LS method, and the proposed method can attain its theoretical variance. Figure 6, Figure 7 and Figure 8 also show that the positioning accuracy can be improved by increasing the number of radars, SNR, and the number of samples.

## 6. Conclusions

Compared with the LS method, a novel localization method based on a two-step WLS estimator is proposed to increase positioning accuracy for a multi-station dual-frequency radars system in this paper. Simulation results verify the proposed method. The effects of SNR and the number of samples are analyzed in the paper. Both the theoretical variance and CRLB for the proposed method are derived in the paper. This paper mainly focuses on a single MT. Multiple-targets localization in a multi-station dual-frequency radars system is still an ongoing issue. Doppler frequency can be used to distinguish different MTs for a single-station radar, whereas Doppler frequencies from the same MT for various radars in a multi-station radars system are different. Thus, it is hardly to distinguish different MTs using Doppler information in our system. Wrong phase match will lead to false target. One possible way for multiple targets localization in a multi-station radars system is a tracking match based on MT localization and tracking methods [17,18,19]. False targets may not form a trajectory, whereas true targets will have clear trajectory. The studies of multiple targets localization and tracking are left for future work.
